# What is a Certified Hernia Center? The Example of the German Hernia Society and German Society of General and Visceral Surgery

**DOI:** 10.3389/fsurg.2014.00026

**Published:** 2014-07-01

**Authors:** Ferdinand Köckerling, Dieter Berger, Johannes O. Jost

**Affiliations:** ^1^Department of Surgery and Center for Minimally Invasive Surgery, Vivantes Hospital Berlin, Academic Teaching Hospital of Charité Medical School, Berlin, Germany; ^2^Department of Visceral, Thoracic and Pediatric Surgery, Hospital Baden-Baden, Baden-Baden, Germany; ^3^German Society of General and Visceral Surgery, Berlin, Germany

**Keywords:** hernia center, certified hernia center, hernia registry, quality assurance, health care, tailored approach

## Abstract

To date, the scientific definition “hernia center” does not exist and this term is being used by hospitals and private institutions as a marketing instrument. Hernia surgery has become increasingly more complex over the past 25 years. Differentiated use of the various techniques in hernia surgery has been adopted as a “tailored approach” program and requires intensive engagement with, and extensive experience of, the entire field of hernia surgery. Therefore, there is a need for hernia centers. A basic requirement for a credible certification process for hernia centers involves definition of requirements and its verification by hernia societies and/or non-profit organizations that are interested in assuring the best possible quality of hernia surgery. At present, there are two processes for certification of hernia centers by hernia societies or non-profit organizations.

## Introduction

If one types the term “hernia center” into Google, 6,560,000 pages are displayed. But in PubMed not a single scientific publication is shown under the keyword “hernia center.” This demonstrates that to date the scientific definition “hernia center” does not exist and that this term is being used by hospitals and private institutions as a marketing instrument to recruit as many patients with hernia diseases as possible.

Hernia surgery has become increasingly more complex over the past 25 years because of the introduction of novel endoscopic, but also conventional, techniques and of the plethora of medicotechnical devices.

“The consideration of herniorrhaphy among most general surgeons has changed. The past thinking of ‘it’s just a hernia’ is passé and has been replaced with the science-based consideration of patient-related factors, patient selection, anatomic application, fixation strength requirements, and healing considerations of biomaterials, as well as truthful and true physical world-based postoperative activity restrictions” (1).

“This realm of greater understanding of abdominal wall problems and their repair has improved patient outcomes and delivered this form of surgery to a true specialty” (1).

Differentiated use of the various techniques has been adopted as a “tailored approach” program and requires intensive engagement with, and extensive experience of, the entire field of hernia surgery. Eighty-two percent of experienced hernia surgeons are employing the “tailored approach” in hernia surgery (2). The overall domain of hernia surgery has become more demanding (3). A comparative study demonstrated that regardless of the surgical technique (open anterior mesh technique, plug technique, open posterior mesh technique, endoscopic technique), the recurrence rate is significantly higher for general surgeons who are not specialists in hernia surgery compared with hernia specialists (*p* < 0.0001) (4).

Therefore, there is a need for hernia centers (5) in which hernia surgery is practiced by specially accredited hernia surgeons who as far as possible master all hernia surgical techniques and play an active role in training and continuing education as well as in the field of science. That requirement is all the more important since each year some 20 million inguinal hernia operations are performed worldwide. In the USA alone, 1 million, and in Germany 350,000 patients undergo some form of hernia surgery (3, 6, 7).

Each recurrence after primary repair of a hernia constitutes an additional economic burden for the healthcare system and can lead to considerable complications for the patient. Onset of postoperative chronic pain secondarily to hernia surgery procedures is a major problem for the patient concerned and for the treating surgeon.

## Outcome Quality is the Most Important Requirement for a Hernia Center

The currently prevailing situation is well summed up by Prof. Robert Fitzgibbons, Creighton University School of Medicine in Omaha. “The flip side, then, is that everyone can say he or she has the best technique or their practice is a hernia center of excellence. There is no board that regulates hernia centers of excellence. It is more or less just hanging out a shingle. Most of the time, it is just a matter of calling yourself a center of excellence” (5).

Therefore, having patients opt for hernia centers whose credentials have not been verified can lead to a situation where hernia surgery procedures are not conducted in conformance with the guidelines of the hernia societies and thus fail to achieve the proven outcome quality.

A basic requirement for a credible certification process for hernia centers involves definition of requirements and their verification by hernia societies and/or non-profit organizations that are interested in assuring the best possible quality of hernia surgery. Besides, the treatment quality actually achieved by the certified center must be ascertained through obligatory participation in a quality assurance program or registry involving follow-up of patients.

Already back in 1965, the Shouldice Clinic in Toronto published follow-up data for its 24,704 patients at that time (8). There are corroborated data to demonstrate that participation in a registry leads to reduced recurrence rates and improved cost efficiency (9, 10). Hernia centers and their outcome quality are verified and evaluated in regular audits by independent experts appointed by hernia surgical societies or non-profit organizations. Re-evaluation is required if any changes are made to personnel in the certified hernia center.

## Certification Processes for Hernia Centers

At present, there are two processes for certification of hernia centers by hernia societies or non-profit-organizations:

1. Certified Center of Excellence in Hernia Surgery (COEHS) by the non-profit organization Surgical Review Corporation (SRC).
2. Certified Hernia Center of the German Hernia Society (DHG) and the German Society of General and Visceral Surgery (DGAV).

Below are listed the requirements to be met by certified hernia centers:

1. Certified COEHS.
  Established in 2003, SRC is an internationally recognized non-profit health care leader committed to advancing the safety, efficacy, and efficiency of technical care worldwide. To achieve its aims, SRC developed a proven methodology that involves two independent initiatives: a rigorous center of excellence program and a central outcomes’ database. This integrated approach exemplifies the concept of the “Hawthorne effect” – over time, program participants improve the quality of their care simply be entering data that will be subject to evaluation.
  A commitment to excellence necessitates that the requirements be comprehensive, research-based, and verified through a rigorous site inspection and review process. SRC works closely together with leaders in hernia surgery and abdominal wall reconstruction to develop the COEHS program and its standards. COEHS is endorsed by the Asia Pacific Hernia Society (APHS).
2. Certified Hernia Center of the German Hernia Society (DHG) and the German Society of General and Visceral Surgery (DGAV) (11).
  The certification process is broken down into three stages to enable as many hospitals and surgery practices as possible to set up a certified hernia center:
  Stage 1: DHG (German Hernia Society) – seal of quality assurance in hernia surgery
  Stage 2: Competence Center for Hernia Surgery
  Stage 3: Reference Center for Hernia Surgery

## Stage 1: DHG – Seal of Quality Assurance in Hernia Surgery

The following requirements must be met before award and permanent possession of the DHG – seal of quality assurance in hernia surgery:

- Surgical treatment of at least 30 hernia patients per year
- Participation in the Herniamed registry (www.herniamed.de)
- Applicant hernia surgeons must be full members of the German Hernia Society (DHG), including the European Hernia Society (EHS), and subscribe to the journal *Hernia* (block membership)

After 30 patients have been entered into the registry, the seal can be awarded. After 1 year, the patients entered into the registry are checked based on the hospital controlling mechanisms or the annual report of the private-practice surgeons. Data on more than 90% of the patients who had undergone hernia surgery must be entered into the registry.

If these prerequisites are met, recertification is done after a further 2 years, taking account of not only the number of operations and perioperative results but also of the follow-up examinations.

The following outcome quality is required up to 30 days postoperatively:

- Total complication rate for inguinal hernia surgery <5%
- Reoperation rate for inguinal hernia surgery <2%
- Reoperation rate for incisional hernia surgery <10%
- Infection/revision rate after open incisional hernia surgery <10%
- Infection/revision rate after laparoscopic incisional hernia surgery <3%

The problem with the required outcome criteria is, that very often the most complicated abdominal wall defects are treated at large hospitals and university settings not routinely performing inguinal hernia repair and that more complex incisional hernias are not treated at the same units, that perform high volume and high quality surgery for less complex hernias. This case mix also of cause has a considerable effect on outcome data as surgical site infections, where some units will present a false low risk and others a false high risk in relation to the limits set by the societies. Therefore, a precise documentation of all risk factors and hernia classifications with influence on the outcome for risk stratification is an important element of the Herniamed registry.

A rate of at least 60% must be achieved for follow-up. This thus amounts to a 3-year certification cycle.

In principle any future higher-grade certification process will be based on the DHG – seal. An application for certification as a Competence Center or Reference Center for Hernia Surgery can be submitted only after the hospital or surgical practice has possessed the “DHG – seal of quality assurance in hernia surgery” for at least 12 months and has already carried out follow-up examinations over a period of 2 months.

## Stage 2: Competence Center for Hernia Surgery

Based on the certification regulations, the Competence Center for Hernia Surgery must meet the following prerequisites:

- For initial certification, the hospital or practice must be in possession of the “DHG – seal of quality assurance in hernia surgery” for at least 12 months and have already carried out the necessary follow-up examinations over a period of 2 months.
- Performance of at least 200 hernia operations per year, of which at least 30 must be incisional hernia operations.
- Special consultations must be offered once weekly for hernia patients.
- Provision must be made for outpatient surgery.
- A treatment regimen for postoperative pain management must be available.
- A morbidity conference must be held at least once monthly.
- Provision must be made for ultrasound examination and commensurate competence demonstrated.
- Documentation of all hernia operations in Herniamed.
- Postoperative and discharge pain intensity must be recorded.
- Evidence of repair of bilateral inguinal hernia in accordance with the guidelines.
- Evidence of 1-year follow-up must be provided for at least 60% of patients.
- The following outcome quality is required up to 30 days postoperatively:
  - Total complication rate for inguinal hernia surgery <5%
  - Reoperation rate for inguinal hernia surgery <2%
  - Reoperation rate for incisional hernia surgery <10%
  - Infection/revision rate after open incisional hernia surgery <10%
  - Infection/revision rate after laparoscopic incisional hernia surgery <3%
- The responsible surgeons must attend at least once yearly one of the following congresses:
  - Annual congress of the German Society of General and Visceral Surgery (DGAV)
  - DGAV annual congress in cooperation with the Gastroenterologist (Visceral Medicine)
  - German Hernia Society (DHG) congresses/symposiums
  - Hernia Surgery Working Group (CAH) congresses/symposiums
  - EHS congresses/symposiums

## Stage 3: Reference Center for Hernia Surgery

The following criteria must be met to obtain certification as a “Reference Center for Hernia Surgery”:

- Evidence of compliance with all requirements for a Competence Center for Hernia Surgery must be provided.
- Performance of at least 250 hernia operations per year, of which at least 50 must be incisional hernia operations, five complex hernias (e.g., parastomal hernia, component separation technique) and five hiatal hernias.
- The Reference Center must have facilities/skills to conduct all laparoscopic/endoscopic and open techniques for hernia operations.
- The Reference Center must enter into a cooperation contract with a plastic surgeon.
- Each year evidence must be produced of presentation of at least two papers or posters at a DHG-sponsored or international hernia congress or of one publication in a peer-reviewed journal.
- A Reference Center makes provision for continuing education seminars and guest visits in the field of hernia surgery. The continuing education seminars must be certified with a total of eight credits per year by the competent Federal Medical Council.

Until March 2014, the German Hernia Society and the German Society of General and Visceral Surgery have certified 286 institutions with the DHG-Seal of Quality Assurance in Hernia Surgery, 18 hospitals as Competence Center for Hernia Surgery, and 3 hospitals as Reference Center for Hernia Surgery.

## Summary

It is now high time to put an end to overuse of the term “hernia center.” Instead, the hernia societies and non-profit organizations committed to optimum quality of hernia surgery are called upon to define clear requirements and certification processes for hernia centers, so that the patient has access to a genuine orientation guide. Modern hernia surgery driven by the “tailored approach” concept has become so demanding that the latter cannot be implemented in all surgical centers. The era of hernia surgery characterized by the motto “it is just a hernia” is long gone and what is needed today is intensive engagement with the topic and commensurate clinical experience. Successful outcomes can be assured in hernia surgery only through consistent implementation of quality requirements. The first steps in that direction have been taken with the Certified COEHS program of the SRC and the Certified Hernia Center program of the German Hernia Society (DHG) and the German Society of General and Visceral Surgery (DGAV). All self-appointed hernia centers should face up to this challenge and become certified. To that effect, hernia societies worldwide must observe the examples listed here.

## Conflict of Interest Statement

The authors declare that the research was conducted in the absence of any commercial or financial relationships that could be construed as a potential conflict of interest.
